# Interactive Virtual Reality versus Vignette-Based Assessment of Children’s Aggressive Social Information Processing

**DOI:** 10.1007/s10802-021-00879-w

**Published:** 2021-10-14

**Authors:** Rogier E. J. Verhoef, Esmée E. Verhulp, Anouk van Dijk, Bram O. de Castro

**Affiliations:** 1 Department of Developmental Psychology, Utrecht University, Heidelberglaan 1, 3508 TC Utrecht, The Netherlands; 2 Research Institute of Child Development and Education, University of Amsterdam, Nieuwe Achtergracht 127, 1001 NG Amsterdam, The Netherlands; 3 Centre for Urban Mental Health, University of Amsterdam, Nieuwe Achtergracht 127, 1001 NG Amsterdam, The Netherlands

**Keywords:** Social information processing, Aggression, Children, Virtual Reality, Reactive and proactive motives

## Abstract

This study examined whether interactive Virtual Reality (VR) provides a more ecologically valid assessment of children’s aggressive social information processing (SIP) and aggressive responses than a standard vignette-based assessment. We developed a virtual classroom where children could meet and play games with virtual peers. Participants were boys (*N* = 184; ages 7–13) from regular education and special education for children with disruptive behavior problems. They reported on their SIP in four scenarios (i.e., two instrumental gain and two provocation scenarios) presented through both interactive VR and vignettes. Teachers reported on children’s real-life aggressive behavior and reactive and proactive motives for aggression. Results demonstrated that children found the interactive VR assessment more emotionally engaging and immersive than the vignette-based assessment. Moreover, compared to vignettes, the interactive VR assessment evoked higher levels of aggressive SIP and responses in provocation scenarios only. Results supported the enhanced predictive validity of the interactive VR assessment of children’s aggressive SIP and responses, which predicted children’s real-life aggression above and beyond the vignette-based assessment with 2 to 12% additional explained variance. Similar results were found for children’s real-life reactive and proactive motives for aggression, with 3 to 12% additional variance explained by interactive VR above and beyond vignettes. Interactive VR did not, however, evoke larger individual differences (i.e., variances) in children’s aggressive SIP and responses than vignettes. Together, these findings suggest that interactive VR provides a more ecologically valid method to assess children’s aggressive SIP and responses than hypothetical vignettes.

**Supplementary Information:**

The online version contains supplementary material available at 10.1007/s10802-021-00879-w.

Children are often confronted with challenging social situations, such as not being allowed to join a peer group or being reprimanded by their teachers or parents. Such situations are likely to elicit strong emotions, which may affect children’s thinking and responding in these situations (Caporaso & Marcovitch, 2021; Lemerise & Arsenio, 2000; Reijntjes et al., 2011). In many children, strong emotions such as anger, frustration, desire, or jealousy may trigger aggressive cognitions that would not have been triggered without these emotions. For instance, children may only interpret others’ behavior as hostile when they feel frustrated, or may only justify stealing when they strongly desire an object. Thus, to better understand, predict, and treat children’s aggressive behavior, we need to assess how children think in social situations *when they are emotionally engaged*. Yet traditional methods to assess children’s social information processing (SIP) often use hypothetical stories (i.e., vignettes) that are unlikely to elicit strong emotions. We have therefore developed an interactive Virtual Reality (VR) environment to assess children’s aggressive SIP and responses. The present study examines whether our VR-based assessment of children’s SIP and responses better predicts their real-life aggressive behavior compared to a standard, vignette-based assessment.

Our interactive VR assessment is based on the SIP model (Crick & Dodge, 1994; Lemerise & Arsenio, 2000). This SIP model proposes that children’s behavioral responses to social situations result from a sequence of mental processing steps: (1) encoding of social cues, (2) representation of social cues, (3) specification of interactional goals, (4) generation of responses, (5) evaluation of responses, and (6) enactment of a selected response. Children’s aggressive behavior has been associated with deviations in each of these SIP steps, such as biased encoding, making hostile intent attributions, setting interactional goals directed at revenge or instrumental gain, generating more aggressive responses, and evaluating aggressive responses and their outcomes more positively (for reviews, see: De Castro & Van Dijk, 2017; Dodge, 2011). Moreover, children with aggressive behavior problems are more likely to experience anger (De Castro & Van Dijk, 2017), and research suggests that their SIP is more strongly affected by negative emotions (De Castro et al., 2003).

Previous work has shown that children’s SIP patterns explain substantial variance in their concurrent and future aggressive behavior (e.g., De Castro & Van Dijk, 2017; Lansford et al., 2006; Verhoef et al., 2019). Nonetheless, findings vary considerably between studies and SIP measures used. A meta-analysis (Verhoef et al., 2019) revealed that the association between aggressive behavior and children’s hostile intent attributions was stronger in studies using actual social interactions (*d* = 1.33) than in studies using vignettes (*d* = 0.23 to 0.44) or video-game tasks (*d* = 0.36; Yaros et al., 2014). The small to moderate effect sizes for vignettes and video games may be due to a lack of emotional engagement (i.e., vignettes may not evoke strong emotions) or limited ecological validity (i.e., video games may not resemble real-life social interaction). These findings align with theoretical work suggesting that strong emotions such as anger or frustration may trigger aggressive SIP patterns that are not triggered when children are calm (Anderson & Bushman, 2002; Lemerise & Arsenio, 2000; Verhoef et al., 2021a). However, few studies exist that used actual social interactions to assess children’s SIP—possibly because using this method is challenging in terms of standardization and ethics (Underwood, 2005).

Ideally, SIP assessment would combine highly emotional engaging, realistic social interactions with adequate standardization and ethically and practically feasible methodology. To attain this goal, we developed an interactive VR classroom where children can walk around freely, talk to virtual peers, and play games, allowing us to present standardized social events within an engaging environment. As children are fully immersed in the VR environment, the peer interactions they have (e.g., their game being ruined by a peer) may evoke substantial levels of anger, frustration, or jealousy. A recent pilot study revealed that our interactive VR assessment evoked larger individual differences in aggressive responses than a vignette-based assessment (Verhoef et al., 2021b), suggesting that interactive VR may also enhance the prediction of individual differences in real-life aggressive behavior. The present study capitalizes on these findings by examining whether our interactive VR assessment of children’s SIP indeed is (1) more immersive and emotionally engaging, and (2) more strongly associated with children’s real-life aggression, compared to a vignette-based assessment of children’s SIP.

Another advantage of using interactive VR may be that it allows for more precise assessment of distinct SIP patterns underlying reactive and proactive aggression (Dodge, 1991). Reactive aggression—an impulsive aggressive response to perceived threat or provocation (Dodge, 1991)—may stem from SIP characterized by excessive anger, heightened sensitivity to threatening cues, a tendency to attribute hostile intent to others, and goals directed at self-defense or taking revenge (e.g., Hubbard et al., 2010; Martinelli et al., 2018). Such reactive SIP patterns may particularly be triggered in provocation contexts (Hubbard et al., 2010) where children are refused to join a peer group (i.e., social provocation) or a peer damages their property (i.e., object provocation). In contrast, proactive aggression—planned aggressive behavior aimed at obtaining a desired outcome (Dodge, 1991)—may stem from SIP characterized by instrumental goals, positive outcome expectations of aggression, and positive evaluations of aggression (e.g., Hubbard et al., 2010). Such proactive SIP patterns may particularly be triggered in instrumental gain contexts (Hubbard et al., 2010), where children have the opportunity to steal something (i.e., object acquisition) or win a game by (i.e., competition). Although reactive and proactive motives for aggression can be mixed (e.g., taking revenge to show who is the boss; Bushman & Anderson, 2001), there is ample empirical work to suggest that they often occur in isolation (Polman et al., 2007; Van Dijk et al., 2021). Earlier studies, however, have not always found clearly delineated reactive versus proactive SIP patterns; possibly because their vignette-based assessment did not evoke the specific emotions underlying real-life reactive and proactive aggression (e.g., Crick & Dodge, 1996; Dodge et al., 1997; Oostermeijer et al., 2016; Stoltz et al., 2013).

Our interactive VR may address this issue by immersing children in engaging social interactions with virtual peers, where they are actually (not just hypothetically) provoked or tempted to use aggression. They may experience anger, frustration, or jealousy, activating the unique SIP patterns underlying reactive- and proactive aggression. Interactive VR then allows children to actually aggress against virtual peers instead of reporting on their hypothetical aggressive responses as with vignettes. Consequently, interactive VR permits an assessment of children’s outcome expectancies and evaluations regarding their actual behavior instead of presenting them with hypothetical response options they might never carry out in real life.

In sum, the present study examines whether interactive VR provides a better assessment of children’s aggressive SIP and responding than a standard, vignette-based assessment. We chose vignettes for this comparison because they are the standard method to assess children’s SIP, and have been shown to yield similarly modest associations with children’s real-life aggression as other methods, such as video-game tasks (Verhoef et al., 2019). Children completed both an interactive VR-based and a hypothetical vignette-based assessment of SIP, and teachers reported on their aggressive behavior. We had three main goals. First, we tested whether interactive VR, compared to vignettes, would elicit higher levels of emotional engagement (1a) and immersion (1b). Consequently, we expected that interactive VR would trigger aggressive SIP and response patterns that are not triggered when children are calm. This should result in larger individual differences (i.e., variances) in SIP and aggressive responses (1c), and higher scores on aggressive SIP and aggressive responses (1d). Moreover, it should result in more congruent SIP and response patterns, visible as stronger correlations between all SIP and aggressive response variables in each scenario (1e). Second, we examined whether interactive VR explained additional variance in children’s real-life aggressive behavior reported by teachers, above and beyond the vignette-based assessment. We examined this both for the assessment of children’s aggressive SIP (2a) and children’s aggressive responses (2b). Third, we examined whether interactive VR explained additional variance in teacher-reported reactive and proactive motives for aggression, above and beyond the vignette-based assessment—again, both for aggressive SIP (3a) and aggressive responses (3b).

## Method

### Participants

Participants were 184 Dutch boys ages 7 to 13 years (*M* = 10.22; *SD* = 1.30). They were recruited from 18 Dutch primary schools. Schools were from neighbourhoods representative of the Dutch population, with on average 9% inhabitants with a Western migration background (*SD* = 3%), 13% with a non-Western migration background (*SD* = 9%), 21% with a lower educational level (*SD* = 4%), and with 7% of the households having a low-income (*SD* = 3%) (Statistics Netherlands, 2018, 2019). To maximize variance in aggressive behavior, boys high on disruptive behavior problems were oversampled by including boys from special education for disruptive behavior problems (*n* = 118) and a random sample of boys from regular education (*n* = 66). In the Netherlands, special education for children with disruptive behavior problems and/or psychiatric problems is reserved for children whose behavior problems are so severe that they require extra support that cannot be provided in regular education. In our study, boys from special education were nominated by their teacher for frequently showing aggressive behavior problems. Boys were excluded if they had an IQ below 80 or an Autism Spectrum Disorder (ASD) according to their casefiles, or had a clinical score on ASD symptoms on the teacher-rated Social Emotional Questionnaire (SEQ; Scholte & Van der Ploeg, 2007). Schools sent parents an information letter in which the study was explained. All parents provided written consent for their child’s participation in the study by signing the attached informed consent form and returning it to their child’s teacher. Boys provided verbal assent.

### Procedure

Participants were individually tested in a silent room at their school by trained graduate students or the first author. Graduate students were trained in multiple sessions by the first author and were supervised during the first two assessments to ensure assessment fidelity. The interactive VR- and vignette-based SIP assessments both lasted 45 min and were completed on two different days with approximately one week in between. We counterbalanced the order of these assessments across participants to control for order effects. At the end of each assessment, boys reported on their emotional engagement and immersion during the assessment. Boys received a small monetary reward (€5) for their participation. Teachers reported on boys’ aggressive behavior and filled out the SEQ through online questionnaires (response rate = 98%). The study was approved by the Medical Ethics Committee of University Medical Center Utrecht.

### Materials

#### Interactive Virtual Reality Environment

Participants wore VR glasses to immerse them in the VR environment. They could walk around freely (in a demarcated 4 × 4 m space), use controllers that mimicked their hands, and respond in similar fashion as in real life: through verbal and physical behavior. The interactive VR environment was designed as a virtual school classroom where participants could interact and play games with virtual peers (for a detailed description of the interactive VR environment, see: Verhoef et al., 2021b). We presented the virtual classroom to participants as an actual classroom where standard behavior rules applied (e.g., respecting other children) and where they would meet real children from other schools who were also participating in the study. In reality, virtual peers were controlled by the experimenter through default movement options and standardized verbal responses.

Participants could play two games: (1) building a tower of blocks as high as possible, and (2) throwing five balls to hit as many cans from a table as possible. We designed our VR assessment around these games to allow for both peer-directed aggression (e.g., hitting, name calling) and property-directed aggression (e.g., knocking over the peer’s tower). To increase participants’ emotional engagement and to provide experimental control over gains and losses, we included high scores and bonuses for participants’ performance during the games (e.g., building a high tower). The instructions, game rules, and score count were displayed on a digital school board, which also explained these matters through standardized verbal instructions.

#### Virtual Reality Scenarios

Participants were presented with six VR scenarios in a fixed order: (1) practice scenario, (2) neutral scenario, (3) object acquisition, (4) competition, (5) social provocation, and (6) object provocation—all centering around one of the games (i.e., the tower or cans game; randomly assigned). The practice VR scenario served to familiarize participants with the VR environment and game rules by practicing the game without any virtual characters present. The neutral scenario served to familiarize participants with the SIP questions by having them play the game while engaging in neutral small talk with a virtual peer, and asking the SIP questions afterwards. Next, participants completed the four experimental scenarios, which we based on taxonomies of problematic situations for children with aggressive behavior problems (Matthys et al., 2001). The first two scenarios involved instrumental gain. In the object acquisition scenario, participants had the opportunity to steal a block or ball from the virtual peer, which would earn them additional points in the game. In the competition scenario, they could win the game and thus earn additional points by sabotaging the virtual peer’s progress in the game (i.e., by knocking over the peer’s tower, ruining the virtual peer’s balls). The last two scenarios involved provocation. In the social provocation scenario, participants were refused to join the game by two virtual peers. In the object provocation scenario, their game was ruined by a virtual peer. As such, the provocations caused them to earn no points. In the two provocation scenarios, participants could not obtain any points by responding aggressively. We expected these provocation scenarios to elicit the strongest emotions, and therefore presented them last to prevent carry-over effects.

#### Hypothetical Vignettes

For the vignette-based SIP assessment, we developed audiotaped vignettes with the exact same content as the VR scenarios (e.g., describing how participants would gain or lose high scores and bonuses), allowing for a clean comparison between assessment methods. We counterbalanced the type of game across participants (i.e., participants who received the tower game in interactive VR, received the cans game with vignettes, and vice versa). As in most vignette procedures, participants were told that they would listen to stories about everyday social situations with peers and were asked to imagine that each story actually happened to them (Verhoef et al., 2019).

### Measures

#### Emotional Engagement

We assessed children’s emotional engagement during the assessment in two ways. First, we used two items immediately after each assessment to directly capture children’s emotional engagement during the assessment, aiming to minimize the effect of memory on their ratings (i.e., “How angry did you feel when something bad happened to you in VR/vignettes?” and “How much did you care when something bad happened to you in VR/vignettes?”). Children responded on a rating scale from 1 (*not at all*) to 10 (*very*). We averaged the two items to create emotional engagement scores for both interactive VR (*r* = 0.83) and vignettes (*r* = 0.67). Second, to allow children to make a comparison between the VR- and vignette-based assessment, we again administered these two items after they had completed both assessments, but then phrased in comparative form (e.g., for the first item: “You have completed both the VR and the stories. How angry did you feel when something bad happened to you in the VR? And in the stories?;” question order was counterbalanced). We again averaged the two items to create emotional engagement scores for interactive VR (*r* = 0.74) and vignettes (*r* = 0.74).

#### Immersion

We assessed children’s immersion during the assessment in two ways. First, we used six items immediately after each assessment, which were adapted from the Dutch translation of the Igroup Presence Questionnaire (Schubert et al., 1999). Two of the six items had low factor loadings (i.e., below 0.60) and were excluded. The four items used were: 1) “I was totally caught up by the events in VR/vignettes;” 2) “I had the feeling that the events in VR/vignettes were actually happening to me;” 3) “During the VR/vignettes it felt like I was actually experiencing the events;” and 4) “The events in VR/vignettes seemed almost real.” Participants rated the items on a scale from 1 (*strongly disagree*) to 5 (*strongly agree*). We averaged across items to create immersion scores for both interactive VR (α = 0.78) and vignettes (α = 0.81).

Second, to allow children to make a comparison between the VR- and vignette-based assessment, we administered one item after they had completed both assessments, but then phrased in comparative form (i.e., “You have participated in both the VR- and vignette-based assessment. How much did you have the feeling that the events in VR were actually happening to you? And in the stories?”). Children responded on a rating scale from 0 (*not at all*) to 10 (*very*).

#### Aggressive SIP and Responses

We assessed participants’ aggressive SIP and responses in two provocation scenarios and two instrumental gain scenarios (both in interactive VR and with vignettes). Initially, we planned to create aggregate SIP and response variables for provocation and instrumental gain contexts. However, we found low correlations for SIP and response variables between the social provocation and object provocation scenario (i.e., ranging from 0.37-0.60 in VR and from 0.27-0.50 with vignettes) and between the object acquisition and competition scenario (i.e., ranging from 0.34-0.58 in VR and from 0.35-0.48 with vignettes), suggesting that aggressive SIP and behavior may be highly situation specific (Dodge et al., 1985; Matthys et al., 2001). Hence, we decided to create variables for children’s SIP and aggressive responses for each scenario separately.

**Interactive VR Assessment.** We assessed participants’ aggressive responses through observation of their behavior in VR, and used self-report to assess their anger, intent attributions, goals, outcome expectancies, and response evaluations at the end of each VR-scenario. In between scenarios, participants kept their VR-glasses on while replying verbally to the experimenter’s questions. For procedural clarity, we assessed all SIP questions following all scenarios, even though we were only interested in proactive SIP in instrumental scenarios (i.e., instrumental goals, outcome expectancies, and response evaluation) and reactive SIP in provocation scenarios (i.e., anger, hostile intent attribution, and revenge goals).

***Anger.*** Anger was assessed using one item following each VR-scenario: “The other boy did [behavior of other boy]. How angry did this make you feel, on a scale from 1, meaning *not at all*, to 10, meaning *very*?”.

***Hostile Intent Attribution.*** Intent attributions were assessed using two items following each VR-scenario: “The other boy did [behavior of other boy]. To what extent did he try to be mean, on a scale from 1, meaning *not at all*, to 10, meaning *very*?” and “To what extent did he try to hinder you, on a scale from 1 to 10?” These two items were moderately to highly correlated within each of the four VR scenarios (*M* = 0.83, *Mdn* = 0.87, range = 0.67-0.90) and were therefore averaged within each VR-scenario.

***Interaction Goals.*** Interaction goals were assessed using one open-ended question following each VR-scenario: “When the other boy did [behavior of other boy], you did [behavior of participant]. What was the reason you did this?” In line with earlier research (De Castro et al., 2012), the first author coded each answer as *revenge goals* (e.g., “to retaliate,” “because I was angry,” “to defend myself”), *instrumental goals* (e.g., “to win the game,” “to show him who’s the boss”), *goals underlying non-aggressive behavior* (e.g., “to become friends,” “to avoid problems”), or *no goals* (e.g., “I don’t know”). A second rater also coded 35% of the transcriptions. Inter-rater reliability was excellent, with Cohen’s κ ranging from 0.85-0.96 across scenarios (*M* = 0.91, *Mdn* = 0.91). Scores for revenge goals were created by assigning 1 to *revenge goals* codes and 0 to other codes. Similarly, scores for instrumental goals were created by assigning 1 to *instrumental goals* codes and 0 to other codes.

***Aggressive Responses*.** We assessed participants’ behavioral responses in interactive VR through observation. A trained research assistant made detailed descriptions of participants’ behavioral responses in each VR-scenario. The first author coded these descriptions into *non-aggressive behavior* (e.g., prosocial behavior, avoidance), *mild aggressive behavior* (e.g., coercion, verbal aggression), and *severe aggressive behavior* (e.g., physical aggression, destructive aggression) following standard coding procedures (De Castro et al., 2005). If multiple codes applied, the highest category was scored. A second rater also coded 35% of the behavioral descriptions. Inter-rater reliability was excellent, with κ ranging from 0.92–1.00 across scenarios (*M* = 0.97, *Mdn* = 0.98). Because frequencies of *mild aggressive behavior* were low or even absent (i.e., 0 to 17% across VR-scenarios and vignettes, *Mdn* = 2%), we created a dichotomous variable by coding *mild* and *severe aggressive behavior* as 1 and *non-aggressive behavior* as 0.

***Outcome Expectancies.*** Outcome expectancies of aggression were assessed using one item following each VR-scenario: “What did you expect would happen when you [behavior of participant]?” We coded only answers of participants who had actually used aggression in that VR-scenario and assigned missing values to other answers. The first author coded each answer as *positive outcome expectancies of aggression* (e.g., “I would win the game”), or *no positive outcome expectancies of aggression* (e.g., “He would dislike me”). A second rater also coded 35% of the transcriptions. Inter-rater reliability was excellent, with κ being 1.00 for each scenario. Scores for positive outcome expectancies of aggression were created by assigning 1 to *positive outcome expectancies of aggression* and 0 to *no positive outcome expectancies of aggression*.

***Response Evaluations.*** Positive evaluations of aggression were assessed using one item following each VR-scenario: “When the other boy did [behavior of other boy], you did [behavior of participant]. To what extent do you approve your behavior on a scale from 1, meaning *not at all*, to 10, meaning *very*?” We only used scores of children who had actually used aggression in that VR-scenario and coded other scores as missing.

Participants’ outcome expectancies and response evaluations of aggression were only scored when they displayed aggressive responses, limiting the number of observations for these variables. Conversely, other SIP variables (i.e., anger, hostile intent attributions, revenge goals and instrumental goals) could be scored irrespectively of whether participants engaged in aggressive responses, yielding full data for these variables (see Table 1 for descriptive statistics of SIP and aggressive response variables).

**Table 1:** Descriptive statistics of SIP variables for each scenario between VR and Vignettes (VIG)

	**Object Acquisition**		**Competition**		**Social Provocation**^**1**^		**Object Provocation**^**2**^	
	VR	VIG	VR	VIG	VR	VIG	VR	VIG
Anger	1.75 (1.78)	1.65 (1.62)	3.13 (2.66)	4.98 (3.09)	5.36 (2.88)	5.13 (2.94)	6.79 (2.76)	7.54 (2.58)
Hostile Intent Attribution	1.47 (1.21)	1.59 (1.21)	2.17 (2.18)	5.00 (3.09)	5.23 (3.08)	4.75 (2.94)	7.35 (2.78)	6.65 (3.32)
Revenge Goals	1 (1)	1 (1)	6 (3)	15 (8)	51 (28)	19 (10)	92 (51)	69 (38)
Instrumental Goals	41 (23)	43 (24)	37 (20)	27 (15)	16 (9)	6 (3)	12 (7)	7 (4)
Aggressive Responses	42 (23)	44 (24)	43 (24)	42 (23)	69 (38)	25 (14)	105 (58)	77 (42)
Outcome Expectancies^*^	10 (24)	14 (32)	18 (42)	3 (7)	2 (3)	0 (0)	0 (0)	5 (6)
Positive Evaluations^*^	5.17 (3.56)	4.32 (2.77)	4.91 (3.71)	4.26 (3.25)	4.04 (3.24)	6.24 (3.38)	5.22 (3.40)	5.49 (3.16)

Columns display means (*M*) and standard deviations (*SD*) of Anger, Hostile Intent Attribution, and Positive Evaluations, and the number (*n*) and proportion (%) of children displaying Revenge Goals, Instrumental Goals, Aggressive Responses, and Positive Outcome Expectancies

^*^Scores only apply to children who displayed an Aggressive Response for this scenario

^1^Based on *n* = 179 because 5 children had missing data in VR/vignettes

^2^Based on *n* = 178 because 6 children had missing data in VR/vignettes

**Vignette Assessment.** Children reported on their SIP following each vignette. We used the same questions and coding schemes as used for the interactive VR-assessment, except that we formulated the questions as hypothetical (e.g., “How angry would you feel…?”) instead of actual (e.g., “How angry were you…?”). The two items assessing intent attributions were averaged within each vignette as they were highly correlated (*M* = 0.80, *Mdn* = 0.81, range = 0.68-0.90). Inter-rater reliability (κ) for open-ended questions was based on 35% of transcriptions and was excellent for both interaction goals (range = 0.81–1.00, *M* = 0.91, *Mdn* = 0.91) and outcome expectancies (range = 0.83–1.00, *M* = 0.94, *Mdn* = 1.00). We assessed participants’ anticipated behavioral responses for each vignette using an open-ended question (i.e., “What would you do if [social event]?”). Inter-rater reliability was based on 35% of the transcriptions and was excellent, with κ ranging from 0.91–1.00 (*M* = 0.94, *Mdn* = 0.93).

#### Real-Life Aggressive Behavior

Teachers completed two questionnaires to assess participants’ aggressive behavior in real life. First, teachers filled out the Aggressive Behavior subscale of the Dutch version of the Teacher Report Form (TRF; Verhulst et al., 1997). They rated 20 items (e.g., “This child threatens others”) on a 3-point Likert scale (*1* = *not true for this child*, 2 = *somewhat true for this child*, or 3 = *very often true for this child*). Scores were averaged across items (α = 0.96). Second, they filled out the Instrument for Reactive and Proactive Aggression (IRPA; Polman et al., 2009). This instrument differentiates between the frequency of aggression on the one hand, and the motives underlying aggression on the other hand. We used the frequency scale to assess children’s real-life aggressive behavior. Teachers rated the frequency of 7 distinct forms of aggressive behavior (i.e., kicking, pushing, hitting, name calling, arguing, gossiping, and doing sneaky things) in the previous month on a 5-point Likert scale (1 = *never,* 2 = *once,* 3 = *weekly,* 4 = *multiple times a week,* 5 = *daily*). Scores on these seven items were averaged (α = 0.90). IRPA frequency scores (*M* = 1.95, *SD* = 0.86) and TRF scores (*M* = 1.67, *SD* = 0.57) were highly correlated (*r* = 0.85). We therefore standardized and averaged them to create a single aggressive behavior score.

#### Reactive & Proactive Motives for Aggression

We assessed reactive and proactive motives for aggression by again using the IRPA (Polman et al., 2009), but this time the motive scales. For each form of aggression rated above 0, teachers rated 3 reactive motives items (e.g., “Because someone teased or upset him”) and 3 proactive motives items (e.g., “To hurt someone or to be mean”) on a 5-point Likert scale (0 = *never,* 1 = *rarely,* 2 = *sometimes,* 3 = *often,* 4 = *always*). For aggression frequency items rated 0, motives scores were missing by design. We calculated reactive and proactive motives scores by averaging across all reactive motives items (i.e., 3 items times 7 forms of aggression; α = 0.94) and all proactive motives items (α = 0.95), respectively. Thus, high scores on reactive (*M* = 2.75, *SD* = 0.94) or proactive (*M* = 2.04, *SD* = 0.84) motives indicate that if participants engaged in aggressive behavior, they often had reactive or proactive motives. The correlation between reactive and proactive motives was non-significant (*r* = 0.14, *p* = 0.075).

### Statistical Analyses

To test our first hypothesis that interactive VR is more engaging than vignettes, we considered five aspects. First, we examined whether interactive VR yielded higher mean levels of emotional engagement than vignettes, using paired *t-*tests. Second, we examined whether participants’ immersion was higher in VR versus vignettes, also using paired *t*-tests. Third, we examined whether VR elicited larger individual differences in aggressive SIP and aggressive responses than vignettes. To this end, we used an adaptation of the Pittman-Morgan test which replaces Pearson’s *r* with Spearman’s rank correlation to account for non-normal data (McCulloch, 1987). Fourth, we examined whether interactive VR yielded higher scores on aggressive SIP and aggressive responses than vignettes, using paired *t-*tests for continuous SIP variables and McNemar’s tests for dichotomous SIP and response variables. Fifth, we examined whether VR yielded stronger correlations among SIP and aggressive responses than vignettes. To do so, we calculated correlations between all SIP and response variables for each scenario using Pearson’s *r*, Pearson’s *π,* and Point-Biserial correlations. Next, we tested for inequality of the obtained correlation matrices using Steiger’s test (1980), which directly compares all elements of two dependent correlation matrices instead of comparing each correlation separately.

To test our second hypothesis that interactive VR assessment of aggressive SIP (2a) and responses (2b) better predicts children’s aggressive behavior in real life compared to vignettes, we examined whether VR explained additional variance in real life aggression above and beyond vignettes, but not vice versa. For aggressive SIP, we conducted two hierarchical regression analyses: the first with vignette-assessed SIP entered at step 1 and VR-assessed SIP at step 2; the second with VR-assessed SIP at step 1 and vignette-assessed SIP at step 2. For aggressive responses, we repeated these analyses with VR- versus vignette-assessed aggressive responses as predictors.

To test our third hypothesis that interactive VR assessment of aggressive SIP (3a) and responses (3b) better predicts children’s reactive and proactive motives underlying their aggressive behavior in real life compared to vignettes, we conducted the same hierarchical regression analyses as used for our second hypothesis, but then with reactive motives as dependent variables for the provocation scenarios and proactive motives as dependent variables for the instrumental gain scenarios.

## Results

### Preliminary Analyses

Table 1 presents the descriptive statistics for all SIP variables in both VR and vignettes. As most SIP variables were skewed, we conducted our analyses using a bootstrapping procedure with bias-corrected accelerated (BCa) 95% confidence intervals (CI) based on 5000 resamples.

Our VR elicited aggressive responses in 23% to 58% of children, depending on the scenario (Table 1). However, few children who responded aggressively in the VR, also responded aggressively in the same scenario in vignettes (i.e., 9 to 32% across scenarios, *Mdn* = 10%; see Supplementary Material Table S1). As a result, we had insufficient data to compare VR versus vignettes on SIP variables that were only assessed if children actually responded aggressively (i.e., positive outcome expectancies and positive evaluations of aggression). We therefore reported descriptive statistics for these two variables (see Table 1) but excluded them from our main analyses.

### Children’s Engagement in Interactive VR Versus Vignettes

#### Emotional Engagement

As predicted, children reported feeling more emotionally engaged in VR (*M* = 5.59, *SD* = 2.74) than with vignettes (*M* = 2.91, *SD* = 2.37), BCa 95% CI [2.23, 3.10], *p* < 0.001,, *d* = 0.92. The same result was found when we asked children about their engagement after they had completed both assessments: their engagement was higher in VR (*M* = 5.68, *SD* = 2.69) than with vignettes (*M* = 3.05, *SD* = 2.16), BCa 95% CI [2.23, 3.04], *p* < 0.001, *d* = 0.96.

#### Immersion

As predicted, children reported feeling more immersed in VR (*M* = 4.18, *SD* = 0.83) than with vignettes (*M* = 2.57, *SD* = 1.07), BCa 95% CI [1.44, 1.77], *p* < 0.001, *d* = 1.45. They also reported feeling more immersed in VR (*M* = 7.96, *SD* = 2.21) than with vignettes (*M* = 3.70, *SD* = 2.37) after they had completed both assessments, BCa 95% CI [3.85, 4.64], *p* < 0.001, *d* = 1.52.

#### Variances in Aggressive SIP and Responses

We found limited support for our hypothesis that VR elicits larger variances in SIP variables and aggressive responses than vignettes. For the object acquisition and competition scenario respectively, we found no difference in variances between VR and vignettes for instrumental goals, *t*(178) < 0.01, *p* = 1.000, and *t*(179) = 1.56, *p* = 0.120, and aggressive responses, *t*(178) < 0.01, *p* = 1.000, and *t*(179) = 0.21, *p* = 0.835. In the social provocation scenario, we did find higher variances in VR versus vignettes for revenge goals, *t*(178) = 4.87, *p* < 0.001, and aggressive responses, *t*(178) = 4.37, *p* < 0.001, but not for anger, *t*(178) = -0.28, *p* = 0.777, and hostile intent attributions, *t*(178) = 0.61, *p* = 0.544. Last, in the object provocation scenario, we found no support for our hypothesis: there were no differences for anger, *t*(177) = 0.91, *p* = 0.364, revenge goals, *t*(177) = 0.33, *p* = 0.738, and aggressive responses, *t*(177) = -0.02, *p* = 0.983, and, contrary to our expectation, larger variances with vignettes versus VR for hostile intent attributions, *t*(177) = -2.08, *p* = 0.039.

#### Levels of Aggressive SIP and Responses

We tested whether VR yielded more aggressive SIP and responses than vignettes (Table 1). Details of the McNemar’s test for dichotomous variables can be found in Supplementary Material Table S1. Our hypothesis was partly supported. For the object acquisition and competition scenarios respectively, we found no differences between VR and vignettes in instrumental goals, *p* = 1.000, *OR* = 1.000, *p* = 0.154, *OR* = 1.67, nor aggressive responses, *p* = 1.000, *OR* = 1.00, *p* = 0.885, *OR* = 1.09. For the social provocation and object provocation scenarios respectively, children showed more hostile intent attributions, BCa 95% CI [0.00, 0.97], *p* = 0.048, *d* = 0.15, BCa 95% CI [0.07, 1.29], *p* = 0.025, *d* = 0.17, revenge goals, *p* < 0.001, *OR* = 4.67, *p* = 0.014, *OR* = 1.85, and aggressive responses, *p* < 0.001, *OR* = 6.63, *p* = 0.001, *OR* = 2.35, in VR than with vignettes. However, we found no differences in anger between VR and vignettes in the social provocation scenario, BCa 95% CI [-0.24, 0.69], *p* = 0.354, *d* = 0.07, and even higher mean levels of anger for vignettes versus VR in the object provocation scenario, BCa 95% CI [-1.21, -0.28], *p* = 0.002, *d* = -0.24.

#### Correlations between Aggressive SIP Variables and Responses

We tested whether correlations among aggressive SIP and response variables were stronger in VR versus vignettes. Table 2 presents all correlations between these variables for each scenario separately. Steiger’s test to compare correlation matrices showed that support for our hypothesis was limited. Steiger’s test revealed that the correlation matrix of aggressive SIP and response variables was significantly higher for VR versus vignettes for the competition scenario, χ^2^(1) = 23.33, *p* < 0.001, but did not significantly differ between VR and vignettes for the object acquisition scenario, χ^2^(1) = 0.03, *p* = 0.862, social provocation scenario, χ^2^(6) = 6.58, *p* = 0.361, and object provocation scenario, χ^2^(6) = 6.46, *p* = 0.374.

**Table 2:** Bivariate Correlations of SIP and response Variables in VR and Vignettes with real-life aggressive behavior and reactive and proactive motives for aggression in instrumental gain scenarios (object acquisition scenario above the diagonal; competition scenario below) and provocation scenarios (social provocation scenario above the diagonal; object provocation scenario below)

**Instrumental gain scenarios**	1	2	3	4	5	6				
1. VR: Instrumental Goals		0.98^*^	0.27^*^	0.29^*^	0.19^*^	0.27^*^				
2. VR: Aggressive Responses	0.91^*^		0.26^*^	0.28^*^	0.20^*^	0.27^*^				
3. Vignette: Instrumental Goals	0.23^*^	0.18^*^		0.99^*^	0.17^*^	0.15				
4. Vignette: Aggressive Responses	0.27^*^	0.24^*^	0.76^*^		0.20^*^	0.16				
5. Real-Life Aggressive Behavior	0.26^*^	0.29^*^	0.17	0.32^*^		0.58^*^				
6. Real-Life Proactive Motives	0.24^*^	0.23^*^	0.14	0.20^*^	0.58^*^					
**Provocation scenarios**	1	2	3	4	5	6	7	8	9	10
1. VR: Anger		0.52^*^	0.41^*^	0.41^*^	0.37^*^	0.40^*^	0.21^*^	0.23^*^	0.12	0.06
2. VR: Hostile Intent Attribution	0.55^*^		0.45^*^	0.36^*^	0.29^*^	0.40^*^	0.10	0.13	0.05	0.14
3. VR: Revenge Goals	0.41^*^	0.40^*^		0.80^*^	0.09	0.17^*^	0.16^*^	0.19^*^	0.34^*^	0.30^*^
4. VR: Aggressive Responses	0.36^*^	0.40^*^	0.86^*^		0.07	0.14	0.16^*^	0.23^*^	0.35^*^	0.29^*^
5. Vignette: Anger	0.30^*^	0.18^*^	0.11	0.10		0.52^*^	0.32^*^	0.41^*^	0.06	-0.12
6. Vignette: Hostile Intent Attribution	0.10	0.12	0.14	0.11	0.45^*^		0.30^*^	0.35^*^	-0.05	-0.21^*^
7. Vignette: Revenge Goals	0.19^*^	0.16^*^	0.18^*^	0.23^*^	0.43^*^	0.37^*^		0.86^*^	0.17^*^	0.05
8. Vignette: Aggressive Responses	0.19^*^	0.19^*^	0.24^*^	0.28^*^	0.39^*^	0.39^*^	0.91^*^		0.23^*^	0.09
8. Real-Life Aggressive Behavior	0.20^*^	0.29^*^	0.27^*^	0.32^*^	0.15	0.07	0.14	0.20^*^		0.46^*^
9. Real-Life Reactive Motives	0.19^*^	0.21^*^	0.29^*^	0.27^*^	-0.04	-0.07	-0.04	0.01	0.46^*^	

Correlations of SIP and responses in Instrumental Gain Scenarios are reported in the upper part of the Table (Object Acquisition Scenario above the Diagonal; Competition Scenario below) and correlations of SIP and Responses in Provocation Scenarios in the lower part of the Table (Social Provocation Scenario above the Diagonal; Object Provocation Scenario below

All correlations including Instrumental Goals, Revenge Goals or Aggressive Responses are point-biserial correlations, all correlations between Instrumental Goals, Revenge Goals and Aggressive Responses used Pearson’s π, and other correlations used Pearson’s r

^*^Indicates that the bootstrap 95% confidence interval did not include zero

In sum, children reported more emotional engagement and immersion in VR than with vignettes. Partial support was found for VR outperforming vignettes on other aspects: It yielded more variance for 2 out of 12 results, higher levels of aggressive SIP and responses for 6 out of 12 results, and stronger correlations for 1 out of 4 results.

### Predicting Real-Life Aggressive Behavior

Tables 3 and 4 present the results of the hierarchical regression analyses of aggressive behavior in real life regressed on aggressive SIP a) and aggressive responses b), first conducted with vignettes in Step 1 and VR in Step 2, and next with VR in Step 1 and vignettes in Step 2. Analyses were conducted for each scenario separately.

**Table 3:** Hierarchical regression analyses of real-life aggression regressed both on instrumental goals and aggressive responses

				**Object acquisition scenario**							**Competition scenario**				
Step	Predictor	β	β SE		95% CI	∆*R*^2^	*df*	*F* change	β	β SE		95% CI	∆*R*^2^	*df*	*F* change
1	Vignette: Instrumental Goals	0.37	0.20		-0.02-0.77	0.03	1,174	4.54^*^	0.40	0.25		-0.07-0.91	0.02	1,175	3.75
2	Vignette: Instrumental Goals	0.27	0.19		-0.10-0.65	0.02	1,173	4.38^*^	0.23	0.25		-0.26-0.75	0.05	1,174	10.27^**^
	VR: Instrumental Goals	0.37	0.20		-0.01-0.75				0.58^**^	0.18		0.21-0.94			
1	VR: Instrumental Goals	0.44^*^	0.20		0.04-0.82	0.04	1,174	6.66^*^	0.63^**^	0.19		0.27–1.01	0.07	1,175	13.02^***^
2	VR: Instrumental Goals	0.37	0.20		-0.01-0.75	0.01	1,173	2.30	0.58^**^	0.19		0.22-0.95	0.01	1,174	1.18
	Vignette: Instrumental Goals	0.27	0.19		-0.10-0.64				0.23	0.25		-0.21-0.72			
1	Vignette: Aggressive Responses	0.44^*^	0.20		0.07-0.83	0.04	1,174	6.86^*^	0.74^***^	0.20		0.36–1.15	0.10	1,175	19.98^***^
2	Vignette: Aggressive Responses	0.34	0.19		-0.02-0.72	0.02	1,173	4.12^*^	0.61^**^	0.19		0.24–1.00	0.05	1,174	9.19^**^
	VR: Aggressive Responses	0.35	0.19		-0.00-0.73				0.50^**^	0.17		0.15-0.84			
1	VR: Aggressive Responses	0.45^*^	0.20		0.08-0.84	0.04	1,174	7.09^**^	0.66^***^	0.18		0.31–1.00	0.08	1,175	16.07^***^
2	VR: Aggressive Responses	0.35	0.19		-0.00-0.72	0.02	1,173	3.90	0.50^**^	0.17		0.17-0.83	0.06	1,174	12.39^***^
	Vignette: Aggressive Responses	0.34	0.19		-0.01-0.71				0.61^**^	0.19		0.24-0.97			

Hierarchical Regression Analyses were run for the Two Instrumental Gain Scenarios separately, both with Vignettes and VR Entered First. Model output is based on a non-bootstrapped procedure whereas output on separate predictors is based on a bootstrapping procedure

^*^ *p* < .05; ^**^ *p* < .01; ^***^ *p* < .001

**Table 4:** Hierarchical regression analyses of real-life aggression regressed both on reactive SIP and aggressive responses

				**Social Provocation**						**Object Provocation**				
Step	Predictor	β	β SE		95% CI	∆*R*^2^	*df*	*F* change	β	β SE	95% CI	∆*R*^2^	*df*	*F* change
1	Vignette: Anger	0.01	0.03		-0.05-0.08	0.05	3,172	2.80^*^	0.05	0.03	-0.01-0.11	0.04	3,171	2.55
	Vignette: Hostile Intent Attribution	-0.04	0.03		-0.10-0.02				-0.00	0.02	-0.05-0.04			
	Vignette: Revenge Goals	0.67^*^	0.31		0.09–1.28				0.22	0.17	-0.10-0.55			
2	Vignette: Anger	0.02	0.03		-0.05-0.08	0.12	3,169	7.85^***^	0.04	0.03	-0.01-0.10	0.09	3,168	5.73^**^
	Vignette: Hostile Intent Attribution	-0.05	0.03		-0.11-0.02				-0.01	0.02	-0.05-0.03			
	Vignette: Revenge Goals	0.50	0.28		-0.03–1.04				0.14	0.17	-0.18-0.47			
	VR: Anger	0.01	0.03		-0.06-0.07				-0.01	0.03	-0.08-0.06			
	VR: Hostile Intent Attribution	-0.03	0.04		-0.10-0.04				0.07^*^	0.03	0.01-0.14			
	VR: Revenge Goals	0.79^***^	0.19		0.42–1.13				0.35^*^	0.16	0.02-0.65			
1	VR: Anger	0.01	0.03		-0.06-0.08	0.13	3,172	8.67^***^	0.01	0.03	-0.06-0.07	0.11	3,171	7.14^***^
	VR: Hostile Intent Attribution	-0.04	0.03		-0.11-0.03				0.07^*^	0.03	0.01-0.13			
	VR: Revenge Goals	0.83^***^	0.19		47–1.18				0.35^*^	0.15	0.05-0.63			
2	VR: Anger	0.01	0.03		-0.06-0.07	0.03	3,169	2.15	-0.01	0.03	-0.08-0.06	0.02	3,168	1.31
	VR: Hostile Intent Attribution	-0.03	0.04		-0.10-0.04				0.07^*^	0.03	0.01-0.13			
	VR: Revenge Goals	0.79^***^	0.19		0.42–1.15				0.35^*^	0.16	0.03-0.63			
	Vignette: Anger	0.02	0.03		-0.05-0.08				0.04	0.03	-0.01-0.09			
	Vignette: Hostile Intent Attribution	-0.05	0.03		-0.12-0.02				-0.01	0.02	-.05-.03			
	Vignette: Revenge Goals	0.50	0.28		-0.04–1.10				0.14	0.17	-0.19-0.49			
1	Vignette: Aggressive Responses	0.68^*^	0.26		0.19–1.21	0.06	1,174	11.12^**^	0.44^**^	0.15	0.16–74	0.05	1,173	9.80^**^
2	Vignette: Aggressive Responses	0.48	0.25		-0.02–1.00	0.09	1,173	18.07^***^	0.30^*^	0.14	0.02-0.58	0.07	1,172	14.04^***^
	VR: Aggressive Responses	0.60^***^	0.15		0.33-0.89				0.53^***^	0.13	0.28-0.79			
1	VR: Aggressive Responses	0.68^***^	0.15		0.38-0.99	0.12	1,174	23.86^***^	0.62^***^	0.13	0.35-0.87	0.10	1,173	19.80^***^
2	VR: Aggressive Responses	0.60^***^	0.15		0.32-0.90	0.03	1,173	5.78^*^	0.53^***^	0.13	0.27-0.79	0.02	1,172	4.39^*^
	Vignette: Aggressive Responses	0.48	0.25		-0.01-0.98				0.30^*^	0.14	0.02-0.58			

Hierarchical Regression Analyses were run for the Two Provocation Scenarios separately, both with Vignettes and VR Entered First. Model output is based on a non-bootstrapped procedure whereas output on separate predictors is based on a bootstrapping procedure

^*^ *p* < .05; ^**^ *p* < .01; ^***^ *p* < .001

#### Aggressive SIP

Children’s aggressive SIP in all four VR scenarios significantly predicted their real-life aggression, with explained variances at Step 1 ranging from 4 to 13% across scenarios. As expected, effects were weaker for vignettes. Children’s aggressive SIP assessed with vignettes significantly predicted their real-life aggression at Step 1 in the object acquisition scenario (*R*^2^ = 0.03) and social provocation scenario (*R*^2^ = 0.05), but not in the competition (*R*^2^ = 0.02) and object provocation scenario (*R*^2^ = 0.04). Turning to the incremental value of VR, we found that VR entered at Step 2 explained significant variance over and above vignettes in all scenarios (i.e., 2% in object acquisition, 5% in competition, 12% in social provocation, and 9% in object provocation). As predicted, vignettes did not explain significant variance over and above VR in any scenario.

#### Aggressive Responses

Children’s aggressive responses in all four VR scenarios significantly predicted their real-life aggression, with explained variances at Step 1 ranging from 4 to 12% across scenarios. Similar effects were found for vignettes, with explained variances at Step 1 ranging from 4 to 10%. Turning to the incremental value of VR, we found that VR entered at Step 2 explained significant variance over and above vignettes in all scenarios (i.e., 2% in object acquisition, 5% in competition, 9% in social provocation, and 7% in object provocation). However, we also found that vignettes at Step 2 explained significant variance over and above VR in three scenarios, with higher levels of explained variance in the competition scenario (i.e., 6%), but lower levels in in social provocation and object provocation scenarios (i.e., 3% and 2%, respectively).

In sum, all eight hierarchical regression analyses regarding children’s real-life aggression supported the incremental value of VR over vignettes, whereas only three analyses supported the reverse.

### Predicting Reactive & Proactive Motives

Next, we conducted the same set of hierarchical regression analyses as for children’s real-life aggressive behavior, in this case predicting children’s reactive and proactive motives for aggression. Detailed results of these analyses are provided in the Supplementary Materials (Table S2 and S3).

#### Aggressive SIP

As predicted, children’s aggressive SIP in all four VR scenarios significantly predicted their reactive and proactive motives in real life, with explained variances at Step 1 ranging from 6 to 10% across scenarios. Effects were less pronounced for vignettes. Children’s aggressive SIP assessed with vignettes significantly predicted their reactive and proactive motives in the object acquisition scenario (*R*^2^ = 0.03) and social provocation scenario (*R*^2^ = 0.06), but not in the competition (*R*^2^ = 0.02) and object provocation scenario (*R*^2^ < 0.01). Turning to the incremental value of VR, we found that VR entered at Step 2 explained significant variance over and above vignettes in all scenarios (i.e., 6% in object acquisition, 5% in competition, 12% in social provocation, and 11% in object provocation). In contrast, we found that vignettes at Step 2 explained significant variance over and above VR only in the social provocation scenario (i.e., 8%).

#### Aggressive Responses

Children’s aggressive responses in all four VR scenarios significantly predicted their reactive and proactive motives in real life, with explained variances at Step 1 ranging from 5 to 9% across scenarios. Effects were weaker for vignettes. Children’s aggressive responses assessed with vignettes significantly predicted their reactive and proactive motives in the object acquisition scenario (*R*^2^ = 0.03) and competition scenario (*R*^2^ = 0.05), but not in the social provocation (*R*^2^ = 0.01) and object provocation scenario (*R*^2^ < 0.01). Turning to the incremental value of VR, we found that VR entered at Step 2 explained significant variance over and above vignettes in all scenarios (i.e., 5% in object acquisition, 3% in competition, 8% in social provocation, and 8% in object provocation). In contrast, we found that vignettes at Step 2 explained significant variance over and above VR only in the competition scenario (i.e., 3%).

In sum, all eight hierarchical regression analyses regarding children’s reactive and proactive motives supported the incremental value of VR over vignettes, whereas only two analyses supported the reverse.

## Discussion

This study tested whether interactive Virtual Reality (VR) provides a more ecologically valid assessment of social information processing (SIP) underlying aggressive behavior in children than a standard vignette-based assessment. In line with expectations, children reported that the interactive VR assessment was more emotionally engaging and immersive than the vignette-based assessment. Moreover, the assessment of children’s aggressive SIP and responses in VR predicted their real-life aggressive behavior and reactive and proactive motives for aggression, above and beyond the vignette assessment.

Interactive VR immerses children in emotionally engaging social interactions and enables them to actually aggress against virtual peers—an important difference with vignettes, which ask children to consider their hypothetical aggressive responses. Accordingly, interactive VR has evoked higher levels of aggressive SIP and responses in children in provocation scenarios, and improved the predictive validity of their assessed aggressive SIP and responses. These findings support the proposition that emotional engagement influences SIP and consequent behavior (Anderson & Bushman, 2002; Lemerise & Arsenio, 2000). Thus, the emotionally engaging nature of our interactive VR assessment seems to have triggered aggressive SIP patterns and responses that may only occur with sufficient emotional engagement.

We expected that the engaging nature of interactive VR would also evoke larger individual differences in children’s aggressive SIP and responses, and stronger correlations between children’s aggressive SIP and responses compared to vignettes. However, interactive VR and vignettes generally evoked similar variances in children’s aggressive SIP and responses, and similar correlations between aggressive SIP steps and responses. Perhaps, our vignettes validly assessed individual differences in children’s “calm” SIP; that is, the way they would reflect on social situations when they do not experience strong emotions. Such “calm” SIP may also differ between children and show similar correlations between children’s SIP and responses as their emotional SIP, but would be less suitable to predict children’s real-life aggression. Indeed, our findings showed that interactive VR yielded incremental predictive value above and beyond the vignette-based assessment in all four scenarios, both for the prediction of children’s real-life aggression (i.e., 2 to 12% additional explained variance) and underlying reactive and proactive motives (i.e., 3 to 12% additional explained variance).

One unexpected pattern in our findings was that interactive VR seemed to outperform vignettes more so for provocation scenarios than for instrumental gain scenarios: the incremental predictive value of VR versus vignettes was larger in provocation scenarios (with 7 to 12% increases in explained variance in children’s real-life aggression) than in instrumental gain scenarios (with 2 to 5% increases in explained variance in children’s real-life aggression), and only in provocation scenarios children showed more aggressive SIP and responses in VR versus vignettes. Although we expected that the engaging nature of interactive VR would enhance children’s proactive aggressive tendencies in instrumental gain scenarios as well (e.g., because the stakes are higher, so they would experience more jealousy or desire), children did not show more proactive SIP and responses in VR versus vignettes. Possibly, the provocation scenarios were more salient than the instrumental gain scenarios, because the instrumental gain of points in the VR constituted no actual gain outside of the game in the real world. As such, it makes sense that the incremental value of interactive VR was the largest for children’s aggressive SIP and aggressive responses in provocation scenarios. In sum, interactive VR seems to yield an improved assessment of both children’s reactive SIP and proactive SIP patterns and responses compared to vignettes, but the difference in favor of interactive VR is the largest when measuring children’s reactive SIP patterns and aggression.

Several findings on separate SIP steps warrant further discussion. First, children’s interactional goals were the strongest SIP predictor of their real-life aggression and underlying motives, and yielded the largest effect sizes for levels of aggressive SIP in VR versus vignettes. Moreover, as children’s revenge goals were strongly correlated with their anger and hostile intent attributions, they were the only significant SIP step predicting children’s real-life aggression and reactive motives for aggression in most analyses. Although such overlap among predictors (i.e., multicollinearity) may seem problematic from a statistical point of view, it does make sense conceptually, because children’s interactional goals seem to be most proximal to their (aggressive) behavior and may often derive from preceding SIP steps such as anger and hostile intent attributions (Crick & Dodge, 1994).

Second, contrary to our predictions, children reported similar levels of anger in interactive VR and vignettes, and even more anger with vignettes in the object provocation scenario. This finding contrasts with our finding that VR is more emotionally engaging than vignettes. However, it may also reveal a potential limitation of vignettes: asking children to reflect on their anticipated anger in a hypothetical situation could lead them to overestimate how they would actually feel. Indeed, research has shown that individuals generally find it difficult to report on anticipated negative affective states and tend to overestimate them (Robinson & Clore, 2002). Although we do not know whether this was actually the case, the stronger correlations of VR- versus vignette-assessed anger with children’s real-life aggression indicate that children are more accurate when reporting on their anger in interactive VR. Perhaps, as in interactive VR children are actually (not just hypothetically) provoked or tempted to use aggression, they may experience emotions more similar to daily life than the anticipated emotions assessed with vignettes.

This study had several strengths. To our knowledge, it is the first empirical study that used interactive VR to assess children’s aggressive SIP and responses and compared its external validity directly to a standard vignette-based assessment of children’s aggressive SIP and responses. Moreover, we maximized clinically meaningful variance in children’s SIP by recruiting boys from both regular and special education for disruptive behavior problems. The use of interactive VR in a sample with substantial variance in children’s SIP allowed us to test important hypotheses concerning the validity of VR-based assessment of SIP.

Our study also had its limitations. First, as few children responded with aggression in the same scenarios in both VR and vignettes, we were not able to analyze whether interactive VR provides an improved assessment of children’s positive outcome expectancies and response evaluations of aggression, as these were assessed only if children had actually aggressed. Consequently, we tested our hypotheses on children’s proactive SIP and responses in instrumental gain scenarios using two variables only (i.e., instrumental goals and aggressive responses). Second, children’s responses were coded for reliability by the first author, who may have been biased because he was aware of the research questions. However, inter-rater agreement with a second coder who was blind to the research questions was excellent, suggesting that this bias was limited. Third, as interactive VR is obviously more time-consuming and costly to develop than vignettes, we were only able to include four assessment scenarios. Given that children may show aggression in various contexts (De Castro & Van Dijk, 2017), it can be assumed that using only four scenarios involving playing games with peers in a school-setting did not cover the broad range of social situations known to evoke aggression in children. Fourth, as children’s SIP and responses were only weakly to moderately associated across scenarios, we conducted our analyses for each scenario separately. Although this finding aligns with empirical research demonstrating that children’s aggression is situation-specific (e.g., De Castro & Van Dijk, 2017; Matthys et al.*,* 2001), it prohibited us from testing how reliable our SIP measurements were per type of scenario. Last, since our study included only boys between 7–13 years with limited diversity in cultural and socio-economic background, findings cannot directly be generalized to girls, older, or younger children, or children from other cultural or socio-economic backgrounds than our sample.

There are both advantages and disadvantages of using interactive VR to assess children’s aggressive SIP and responses. One important disadvantage is that interactive nature of VR makes establishing ambiguity of social situations more difficult than with vignettes. This interactive nature might enhance the experience of an actual social interaction, however it might also affect ambiguity to some extent (e.g., children who talked a lot with the virtual peer during the interaction might be prone to attribute non-hostile intent). Moreover, interactive VR is obviously costly and time-consuming to develop, and so it is relevant to directly compare this method to other assessment methods besides vignettes, such as video game tasks (e.g., Yaros et al., 2014).

That said, VR has multiple advantages over the use of vignettes. In interactive VR, children are actually provoked, tempted to use aggression, and able to aggress against virtual peers, and may therefore experience similar emotions as in real-life (e.g., anger, frustration), activating similar SIP patterns and responses as in real-world interactions. As such, researchers may examine the effect of a broad range of emotions on children’s SIP; that is, not only anger or frustration, but also shame, guilt, fear, desire or sadness. Relatedly, since children actually ‘respond’ in VR, it is possible to include physiological indicators of children’s arousal, permitting researchers to test more specific hypotheses on the role of emotional arousal in children’s SIP and responses. Moreover, the large experimental control over social stimuli provided by interactive VR (e.g., control over virtual peers’ nonverbal behaviors and emotional expressions) allows researchers to test more specific hypotheses about causal effects of subtle social cues on children’s SIP and responses than has been feasible thus far. In addition, interactive VR may allow researchers to use a broad variety of emotionally engaging contexts known to evoke aggression in children. For example, researchers may present children with more salient cues to evoke proactive SIP and aggression (e.g., by allowing children to obtain actual gains outside of the VR environment). Relatedly, researchers may also examine children’s SIP in other settings than playing games with peers, such as settings with parents, settings which do not involve play, settings that allow for the assessment of relational aggression (e.g., spreading rumors), or settings that better allow to examine cooperative behaviors. Last, using interactive VR may minimize cognitive load), increasing the validity of children’s reported SIP.

In sum, this empirical study demonstrated that interactive VR is an improved method to assess children’s aggressive SIP and behavior compared to a standard vignette-based assessment. The use of VR allows researchers and practitioners to assess aggressive SIP patterns in an emotionally engaging, ecologically valid context that is truly interactive and realistic. Ultimately, interactive VR may also facilitate interventions with children, because it allows for extensive practice with the specific situations relevant to their individual needs, with precise control to adapt difficulty and complexity during the intervention. Moreover, practitioners may use cooperative contexts that yield rewards for specific desirable behaviors (e.g., prosocial), reinforcing these behaviors repeatedly through operant conditioning. As such, interactive VR may further our understanding of the SIP mechanisms underlying aggressive behavior problems in children and may enhance assessment and intervention for children with aggressive behavior problems.

## Supplementary Information

Below is the link to the electronic supplementary material.

Supplementary file1 (DOCX 32 KB)

## Data Availability

The data that support the findings of this study are available through the Open Science Framework at https://doi.org/10.17605/OSF.IO/7SA6M

## Code Availability

The syntax of the analyses run for this study are available through the Open Science Framework (see link above).

## References

[CR1] Anderson CA, Bushman BJ (2002). Human aggression. Annual Review of Psychology.

[CR2] Bushman BJ, Anderson CA (2001). Is it time to pull the plug on hostile versus instrumental aggression dichotomy?. Psychological Review.

[CR3] Caporaso JS, Marcovitch S (2021). The effect of taxing situations on preschool children’s responses to peer conflict. Cognitive Development.

[CR4] Crick NR, Dodge KA (1994). A review and reformulation of social-information-processing mechanisms in children's social adjustment. Psychological Bulletin.

[CR5] Crick NC, Dodge KA (1996). Social information processing deficits in reactive and proactive aggression. Child Development.

[CR6] De Castro, B. O., & Van Dijk, A. (2017). “It’s gonna end up with a fight anyway”: Social cognitive processes in children with disruptive behavior disorders. In: Lochman, J. E., & Matthys, W. (Eds.), *The Wiley handbook of disruptive and impulse-control disorders* (pp. 237–253). John Wiley & Sons Limited. 10.1002/9781119092254.ch15

[CR7] De Castro BO, Merk W, Koops W, Veerman JW, Bosch JD (2005). Emotions in social information processing and their relations with reactive and proactive aggression in referred aggressive boys. Journal of Clinical Child and Adolescent Psychology.

[CR8] De Castro BO, Slot NW, Bosch JD, Koops W, Veerman JW (2003). Negative feelings exacerbate hostile attributions of intent in aggressive boys. Journal of Clinical Child and Adolescent Psychology.

[CR9] De Castro B, Verhulp EE, Runions K (2012). Rage and revenge: Highly aggressive boys’ explanations for their responses to ambiguous provocation. European Journal of Developmental Psychology.

[CR10] Dodge KA, Pepler D, Rubin KH (1991). The structure and function of reactive and proactive aggression. The development and treatment of childhood aggression.

[CR11] Dodge KA, Shaver P, Mikulincer M (2011). Social information processing patterns as mediators of the interaction between genetic factors and life experiences in the development of aggressive behavior. Human aggression and violence: Causes, manifestations, and consequences.

[CR12] Dodge KA, Lochman JE, Harnish JD, Bates JE, Pettit GS (1997). Reactive and proactive aggression in school children and psychiatrically impaired chronically assaultive youth. Journal of Abnormal Psychology.

[CR13] Dodge KA, McClaskey CL, Feldman E (1985). Situational approach to the assessment of social competence in children. Journal of Consulting and Clinical Psychology.

[CR14] Hubbard, J. A., McAuliffe, M. D., Morrow, M. T., & Romano, L. J. (2010). Reactive and proactive aggression in childhood and adolescence: Precursors, outcomes, processes, experiences, and measurement. *Journal of Personality, 78*(1), 95–118. 10.1111/j.1467–6494.2009.00610.x 20433614

[CR15] Lansford JE, Malone PS, Dodge KA, Crozier JC, Pettit GS, Bates JE (2006). A 12-year prospective study of patterns of social information processing problems and externalizing behaviors. Journal of Abnormal Child Psychology.

[CR16] Lemerise EA, Arsenio WF (2000). An integrated model of emotion processes and cognition in social information processing. Child Development.

[CR17] Martinelli A, Ackermann K, Bernhard A, Freitag CM, Schwenck C (2018). Hostile attribution bias and aggression in children and adolescents: A systematic literature review on the influence of aggression subtype and gender. Aggression and Violent Behavior.

[CR18] Matthys W, Maassen GH, Cuperus JM, van Engeland H (2001). The assessment of the situational specificity of children's problem behaviour in peer–peer context. Journal of Child Psychology and Psychiatry.

[CR19] McCulloch CE (1987). Tests for equality of variances with paired data. Communications in Statistics - Theory and Methods.

[CR20] Oostermeijer S, Nieuwenhuijzen M, van de Ven PM, Popma A, Jansen LM (2016). Social information processing problems related to reactive and proactive aggression of adolescents in residential treatment. Personality and Individual Differences.

[CR21] Polman H, de Castro BO, Koops W, van Boxtel HW, Merk WW (2007). A meta-analysis of the distinction between reactive and proactive aggression in children and adolescents. Journal of Abnormal Child Psychology.

[CR22] Polman H, Orobio de Castro B, Thomaes S, van Aken M (2009). New directions in measuring reactive and proactive aggression: Validation of a teacher questionnaire. Journal of Abnormal Child Psychology.

[CR23] Reijntjes A, Thomaes S, Kamphuis J, Bushman B, De Castro BO, Teich M (2011). Explaining the paradoxical rejection-aggression link: The mediating effects of hostile intent attributions, anger, and decreases in state self-esteem on peer rejection-induced aggression in youth. Personality and Social Psychology Bulletin.

[CR24] Robinson MD, Clore GL (2002). Belief and feeling: Evidence for an accessibility model of emotional self-report. Psychological Bulletin.

[CR25] Scholte EM, van der Ploeg JD (2007). Handleiding Sociaal-Emotionele Vragenlijst (SEV).

[CR26] Schubert T, Friedmann F, Regenbrecht H, Paton R, Neilson I (1999). Embodied presence in virtual environments. Visual representations and interpretations.

[CR27] Statistics Netherlands (2018, 2019). *StatLine.* Retrieved June 24, 2021, from https://www.opendata.cbs.nl

[CR28] Steiger JH (1980). Tests for comparing elements of a correlation matrix. Psychological Bulletin.

[CR29] Stoltz S, van Londen M, Dekovic M, De Castro BO, Prinzie P, Lochman JE (2013). Effectiveness of an individual school-based intervention for children with aggressive behaviour: A randomized controlled trial. Behavioural and Cognitive Psychotherapy.

[CR30] Underwood, M. K. (2005). Observing anger and aggression among preadolescent girls and boys: Ethical dilemmas and practical solutions. *Ethics and Behavior, 15*(3), 235–245. 10.1207/s15327019eb1503_4 10.1207/s15327019eb1503_4 16523559

[CR31] Van Dijk A, Hubbard JA, Deschamps PKH, Hiemstra W, Polman H (2021). Do distinct groups of reactively and proactively aggressive children exist?.

[CR32] Verhoef REJ, Alsem SC, Verhulp EE, de Castro BO (2019). Hostile intent attribution and aggressive behavior in children revisited: A meta-analysis. Child Development.

[CR33] Verhoef REJ, Van Dijk A, De Castro BO (2021). A Dual Mode Social Information Processing Model to Explain Individual Differences in Children’s Aggressive Behavior. Clinical Psychological Science, Advance Online Publication..

[CR34] Verhoef REJ, Van Dijk A, Verhulp EE, De Castro BO (2021). Interactive virtual reality assessment of aggressive social information processing in boys with behaviour problems: A pilot study. Clinical Psychology & Psychotherapy.

[CR35] Verhulst, F. C., Van Der Ende, J., & Koot, H. M. (1997). *Handleiding voor de Teachers's Report Form (TRF)*. Rotterdam: afd. Kinder- en jeugdpsychiatrie, Sophia Kinderziekenhuis/ AZR/ EUR

[CR36] Yaros A, Lochman JE, Rosenbaum J, Jimenex-Camargo LA (2014). Real-time hostile attribution measurement and aggression in children. Aggressive Behavior.

