# Supplementary material for: Interactive Virtual Reality versus Vignette-Based Assessment of Children’s Aggressive Social Information Processing
Source: Res Child Adolesc Psychopathol. 2021 Oct 14;50(5):621–36. doi: 10.1007/s10802-021-00879-w (PMC9054903; doi:10.1007/s10802-021-00879-w)
Supplement: Supplementary file 1 — Supplementary file1 (DOCX 32 KB) [file 10802_2021_879_MOESM1_ESM.docx]

**Supplementary Materials**

**Table S1**

*Cross-tabulations of Dichotomous SIP and Response Variables per Scenario for VR and Vignettes.*

|  |  | **Object Acquisition** | | **Competition** | | **Social Provocation** | | **Object Provocation** | |
| --- | --- | --- | --- | --- | --- | --- | --- | --- | --- |
|  |  | Vignette | | Vignette | | Vignette | | Vignette | |
|  | VR | No | Yes | No | Yes | No | Yes | No | Yes |
| Revenge Goals | No | 177 (98.9%) | 1 (0.6%) | 161 (89.4%) | 13 (7.2%) | 119 (66.5%) | 9 (5.0%) | 62 (34.8%) | 26 (14.6%) |
|  | Yes | 1 (0.6%) | 0 (0.0%) | 5 (2.8%) | 1 (0.6%) | 42 (23.5%) | 9 (5.0%) | 48 (27.0%) | 42 (23.6%) |
| Instrumental Goals | No | 115 (64.2%) | 23 (12.8%) | 129 (71.7%) | 15 (8.3%) | 159 (88.8%) | 4 (2.2%) | 159 (89.3%) | 7 (3.9%) |
|  | Yes | 23 (12.8%) | 18 (10.1%) | 25 (13.9%) | 11 (6.1%) | 14 (7.8%) | 2 (1.1%) | 12 (6.7%) | 0 (0.0%) |
| Aggressive  Responses | No | 114 (63.7%) | 23 (12.8%) | 115 (63.9%) | 23 (12.8%) | 102 (57.0%) | 8 (4.5%) | 55 (30.9%) | 20 (11.2%) |
|  | Yes | 23 (12.8%) | 19 (10.6%) | 25 (13.9%) | 17 (9.4%) | 53 (29.6%) | 16 (8.9%) | 47 (26.4%) | 56 (31.5%) |
| Positive Outcomes  of Aggression | No | 10 (52.6%) | 4 (21.1%) | 9 (52.9%) | 0 (0.0%) | 15 (93.8%) | 0 (0.0%) | 52 (92.9%) | 4 (7.1%) |
|  | Yes | 4 (21.1%) | 1 (5.3%) | 8 (47.1%) | 0 (0.0%) | 1 (6.3%) | 0 (0.0%) | 0 (0.0%) | 0 (0.0%) |

*Note.* Odds Ratios in the Main Manuscript are Calculated through the Ratio of Discordant Cells.

**Table S2**

*Hierarchical Regression Analyses of Proactive Motives in Real Life Regressed on both Instrumental Goals and Aggressive Responses.*

|  |  |  | | **Object acquisition** | | | | |  | **Competition** |  |  |  |  |  |
| --- | --- | --- | --- | --- | --- | --- | --- | --- | --- | --- | --- | --- | --- | --- | --- |
| Step | Predictor | β | β SE | | 95% CI | ∆R^2^ | *df* | F change |  | β | β SE | 95% CI | ∆R^2^ | *df* | F change |
| 1 | Vignette: Instrumental Goals | .33 | .17 | | .00-.68 | .03 | 1,157 | 4.59^*^ |  | .33 | .22 | -.08-.76 | .02 | 1,157 | 3.00 |
| 2 | Vignette: Instrumental Goals | .22 | .17 | | -.11-.55 | .06 | 1,156 | 10.06^**^ |  | .20 | .22 | -.21-.64 | .05 | 1,156 | 7.65^**^ |
|  | VR: Instrumental Goals | .50^**^ | .17 | | .16-.84 |  |  |  |  | .45^**^ | .18 | .10-.78 |  |  |  |
| 1 | VR: Instrumental Goals | .55^**^ | .16 | | .23-.86 | .08 | 1,157 | 12.90^***^ |  | .49^**^ | .17 | .16-.83 | .06 | 1,157 | 9.63^**^ |
| 2 | VR: Instrumental Goals | .50^**^ | .17 | | .17-.81 | .01 | 1,156 | 1.95 |  | .45^*^ | .18 | .10-.80 | .01 | 1,156 | 1.14 |
|  | Vignette: Instrumental Goals | .22 | .17 | | -.10-.55 |  |  |  |  | .20 | .22 | -.20-.64 |  |  |  |
| 1 | Vignette: Aggressive Responses | .34^*^ | .17 | | .01-.69 | .03 | 1,157 | 4.96^*^ |  | .43^**^ | .15 | .15-.72 | .05 | 1,157 | 7.92^**^ |
| 2 | Vignette: Aggressive Responses | .22 | .17 | | -.11-.57 | .05 | 1,156 | 9.25^**^ |  | .34^*^ | .15 | .06-.64 | .03 | 1,156 | 5.74^*^ |
|  | VR: Aggressive Responses | .47^**^ | .17 | | .16-.80 |  |  |  |  | .36^*^ | .16 | .05-.68 |  |  |  |
| 1 | VR: Aggressive Responses | .53^**^ | .16 | | .20-.86 | .07 | 1,157 | 12.28^**^ |  | .44^**^ | .16 | .14-.76 | .05 | 1,157 | 8.86^**^ |
| 2 | VR: Aggressive Responses | .47^**^ | .17 | | .14-.81 | .01 | 1,156 | 2.11 |  | .36^*^ | .16 | .05-.69 | .03 | 1,156 | 4.82^*^ |
|  | Vignette: Aggressive Responses | .22 | .17 | | -.09-.54 |  |  |  |  | .34^*^ | .16 | .05-.65 |  |  |  |

*Note.* Hierarchical Regression Analyses were run for the Two Instrumental Gain Scenarios separately, both with Vignettes and VR Entered First. Model output is based on a non-bootstrapped procedure whereas output on separate predictors is based on a bootstrapping procedure.

^*^ *p* < .05; ^**^ *p* <.01; ^***^ *p* <.001.

**Table S3**

*Hierarchical Regression Analyses of Reactive Motives in Real Life Regressed on both Reactive SIP and Aggressive Responses.*

|  |  |  | | **Social Provocation** | | | | |  |  | **Object Provocation** | | | | |
| --- | --- | --- | --- | --- | --- | --- | --- | --- | --- | --- | --- | --- | --- | --- | --- |
| Step | Predictor | β | β SE | | 95% CI | ∆*R*^2^ | *df* | *F* change |  | β | β SE | 95% CI | ∆*R*^2^ | *df* | *F* change |
| 1 | Vignette: Anger | -.03 | .03 | | -.09-.04 | .06 | 3,154 | 3.18^*^ |  | .00 | .04 | -.06-.07 | < .01 | 3,153 | 0.21 |
|  | Vignette: Hostile Intent Attribution | -.06^*^ | .03 | | -.12--.01 |  |  |  |  | -.02 | .03 | -.07-.03 |  |  |  |
|  | Vignette: Revenge Goals | .46 | .24 | | .00-.93 |  |  |  |  | -00 | .18 | -.34-.34 |  |  |  |
| 2 | Vignette: Anger | -.03 | .03 | | -.09-.04 | .12 | 3,151 | 7.55^***^ |  | -.01 | .03 | -.08-.05 | .11 | 3,150 | 6.41^***^ |
|  | Vignette: Hostile Intent Attribution | -.09^**^ | .03 | | -.15--.04 |  |  |  |  | -.02 | .02 | -.07-.03 |  |  |  |
|  | Vignette: Revenge Goals | .37 | .24 | | -.08-.85 |  |  |  |  | -.10 | .18 | -.43-.23 |  |  |  |
|  | VR: Anger | -.00 | .03 | | -.07-.06 |  |  |  |  | .02 | .03 | -.05-.08 |  |  |  |
|  | VR: Hostile Intent Attribution | .04 | .03 | | -.02-.10 |  |  |  |  | .04 | .03 | -.03-.11 |  |  |  |
|  | VR: Revenge Goals | .58^**^ | .18 | | .22-.94 |  |  |  |  | .48^**^ | .16 | .16-.80 |  |  |  |
| 1 | VR: Anger | -.04 | .03 | | -.09-.03 | .10 | 3,154 | 5.65^**^ |  | .01 | .03 | -.05-.07 | .10 | 3,153 | 5.87^**^ |
|  | VR: Hostile Intent Attribution | .01 | .03 | | -.04-.06 |  |  |  |  | .04 | .03 | -.03-.10 |  |  |  |
|  | VR: Revenge Goals | .66^**^ | .18 | | .32-.1.03 |  |  |  |  | .46 ^**^ | .16 | .15-.77 |  |  |  |
| 2 | VR: Anger | -.00 | .03 | | -.07-.06 | .08 | 3,151 | 5.04^**^ |  | .02 | .03 | -.04-.08 | .01 | 3,150 | 0.79 |
|  | VR: Hostile Intent Attribution | .04 | .03 | | -.02-.10 |  |  |  |  | .04 | .04 | -.03-.11 |  |  |  |
|  | VR: Revenge Goals | .58^**^ | .18 | | .22-.94 |  |  |  |  | .48^**^ | .16 | .18-.79 |  |  |  |
|  | Vignette: Anger | -.03 | .03 | | -.09-.04 |  |  |  |  | -.01 | .03 | -.07-.05 |  |  |  |
|  | Vignette: Hostile Intent Attribution | -.09^**^ | .03 | | -.15--.04 |  |  |  |  | -.02 | .03 | -.07-.03 |  |  |  |
|  | Vignette: Revenge Goals | .37 | .24 | | -.08-.85 |  |  |  |  | -.10 | .18 | -.43-.22 |  |  |  |
| 1 | Vignette: Aggressive Responses | .30 | .20 | | -.09-70 | .01 | 1,156 | 2.01 |  | .07 | .15 | -.23-.36 | < .01 | 1,154 | 0.18 |
| 2 | Vignette: Aggressive Responses | .15 | .22 | | -.28-.59 | .08 | 1,155 | 12.91^***^ |  | -.10 | .16 | -.42-.22 | .08 | 1,151 | 13.20^***^ |
|  | VR: Aggressive Responses | .54^**^ | .15 | | .24-.84 |  |  |  |  | .57^**^ | .17 | .24-.89 |  |  |  |
| 1 | VR: Aggressive Responses | .56^***^ | .15 | | .27-.84 | .09 | 1,156 | 14.06^***^ |  | .54^**^ | .15 | .24-.84 | .08 | 1,154 | 13.04^***^ |
| 2 | VR: Aggressive Responses | .54^**^ | .16 | | .23-.84 | < .01 | 1,155 | 0.51 |  | .57^**^ | .16 | .24-.89 | < .01 | 1,151 | 0.40 |
|  | Vignette: Aggressive Responses | .15 | .21 | | -.29-.59 |  |  |  |  | -.10 | .16 | -.41-.19 |  |  |  |

^*^ *p* < .05; ^**^ *p* <.01; ^***^ *p* <.001.
